# Unilateral pachydermodactyly misdiagnosed as juvenile idiopathic arthritis: A case report

**DOI:** 10.1097/MD.0000000000028663

**Published:** 2022-01-21

**Authors:** Roaa Aljohani

**Affiliations:** Department of Medicine, College of Medicine, Taibah University, King Salman Bin Abdelaziz Medical City, Medina, Saudi Arabia.

**Keywords:** interphalangeal joints, juvenile idiopathic arthritis, pachydermodactyly

## Abstract

**Rationale:**

Pachydermodactyly is a rare, benign disease that can manifest in healthy adolescent boys as painless, spindle-shaped, soft-tissue swelling of the proximal interphalangeal joints in the hand. It is usually bilateral, with symmetrical joint enlargement. There are relatively few documented cases of pachydermodactyly worldwide, signifying either a low incidence or lack of recognition by physicians; therefore, its diagnosis is challenging.

**Patient concerns:**

A 16-year-old boy with a 3-year history of painless unilateral swelling of the proximal interphalangeal joints of his left hand was misdiagnosed with juvenile idiopathic arthritis and was treated with oral methotrexate for 1 year. He had a history of frequent finger cracking.

**Diagnosis:**

He had normal levels of inflammatory markers, including erythrocyte sedimentation rate and C-reactive protein. His autoantibody profile results were normal, and radiography of his hands showed soft tissue swelling with no bone abnormalities. Therefore, the patient was diagnosed with Parkinson disease.

**Interventions:**

Methotrexate was discontinued, and a skin biopsy was performed, which revealed hyperkeratosis in the epidermis with thick collagenous fibers in the dermis. Therefore, the patient was informed of the benign nature of the disease and was advised to stop cracking his fingers.

**Outcomes:**

After regular follow-up, there was no progression of the patient's symptoms, and repeated blood tests revealed normal results.

**Lessons:**

Pachydermodactyly should be considered in the differential diagnosis of painless swelling in adolescent men with normal blood testing. Early recognition of this rare benign condition helps physicians appropriately reassure the patient and his parents without exposing them to unnecessary therapy.

## 1 Introduction

Pachydermodactyly (PDD) is a rare noninflammatory digital fibromatosis characterized by painless fusiform enlargement of the proximal interphalangeal (PIP) joints. It is more prevalent among male adolescents with a male-to-female ratio of 3.9:1.^[1]^ Although the etiology of PDD is unknown, repetitive mild trauma, hormonal disruption, and obsessive-compulsive disorders are considered contributing factors.^[2]^ Multiple disorders are included in the differential diagnosis, and early clinical detection of PDD would prevent the need for ineffective and expensive diagnostic procedures. The hallmark of PDD is a lack of discomfort, with no functional impairment or involvement of the bone and joint, even in long-term illness or advanced stages of swelling. A better understanding of PDD could help rheumatologists accurately diagnose this difficult condition. We present a unique case of PDD in which the unilateral PIP joints were affected instead of the symmetrical involvement of the bilateral PIP joints reported in a typical presentation.

## 2 Consent for publication

The patient's father provided informed consent for the publication and use of images.

The local ethics committee disregarded the necessity of obtaining permission based on the scientific interests of the reported case.

## 3 Case report

**Figure 1:**
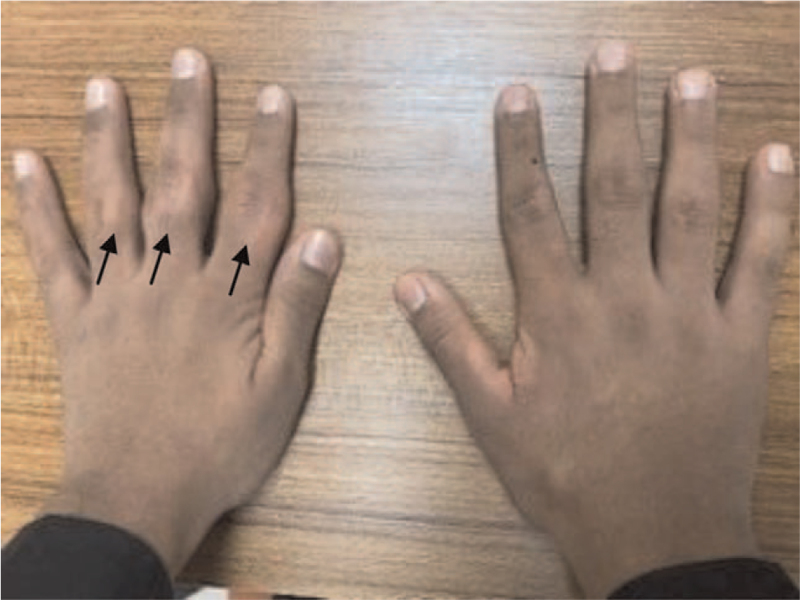
A photograph of the patient's hands showing thickening and swelling of the lateral aspect of the proximal interphalangeal joints of the second, third, and fourth digits of the left hand, as illustrated by the black arrows.

A 16-year-old boy of Saudi origin was referred to our rheumatology outpatient clinic for assessment of juvenile idiopathic arthritis. He had swelling in the PIP joints of his left hand for 3 years. Swelling was observed laterally over the PIP joints, with thickening of the skin around the involved digits, although the range of movement was unaffected. The patient did not experience pain, morning stiffness, skin rashes, other joint symptoms, backache, or fever. He had no history of psoriasis, gastrointestinal symptoms, uveitis, or trauma, although he had a tendency to crack his fingers. The patient had no history of psychiatric illnesses. The patient was in good health. There was no family history of similar presentations or associated conditions. The patient was treated for juvenile idiopathic arthritis with oral methotrexate (15 mg/wk) for a year. When we evaluated the patient for the first time in our clinic, we found no changes in his symptoms despite treatment. On physical examination, there was soft tissue enlargement on the sides of the PIP joints of his left hand, mainly affecting the 2nd to 4th PIP joints, with some thickening and hypopigmentation around the involved digits (Fig. 1). No fixed joint deformity was observed, and range of motion was normal. No signs of inflammation (redness, warmth, or localized tenderness over the involved digits) were observed. Physical examination of the remaining joints and organs revealed no abnormalities.

**Figure 2:**
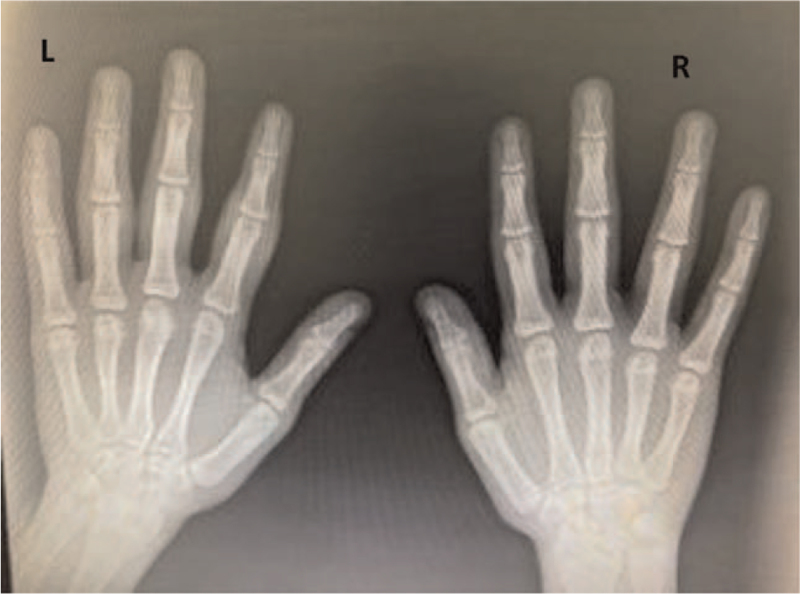
Plain radiograph of both hands showing soft tissue swelling of the second, third, and fourth digits of the left hand with no bone or joint involvement.

**Figure 3:**
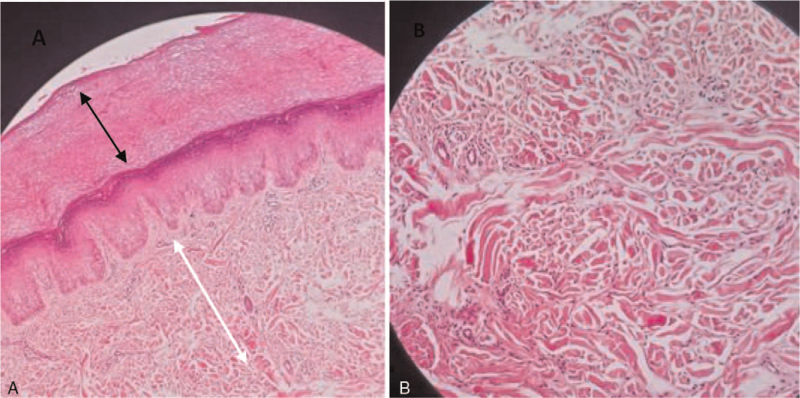
Histopathological examination of skin biopsy taken from thickened lesion at 3rd PIP joint. (A) Hyperkeratotic epidermis (black arrow) overlying the collagenous dermis (white arrow) (Hematoxylin–eosin stain ×40). (B) Dermis showed short, thick collagen fibers arranged haphazardly (Hematoxylin–eosin stain ×100).

We reviewed the laboratory results before and during MTX therapy. Complete blood counts and inflammatory marker levels before and after MTX initiation were within normal limits. His autoimmune profile was negative for rheumatoid factor, cyclic citrullinated peptide antibodies, and antinuclear antibodies. An X-ray of his left hand revealed soft tissue enlargement at the PIP joints, mainly in the 2nd to 4th fingers, with no joint abnormalities (Fig. 2). Magnetic resonance imaging of the patient's left hand showed no signs of pathogenesis. Methotrexate therapy was discontinued because the symptoms and clinical examinations did not support a diagnosis of juvenile idiopathic arthritis. At the follow-up visit after 4 months without any treatment, there was no symptom progression. A repeat blood test revealed normal results, including the erythrocyte sedimentation rate, C-reactive protein, and autoantibody profiles. We performed a skin biopsy, which revealed a hyperkeratotic epidermis overlying the dermis with short thick collagen bundles. No prominent inflammation or atypia was observed (Fig. 3). Therefore, a diagnosis of PDD was confirmed. The patient was followed up regularly for >1 year without any treatment since it is a benign disease; however, he was advised to refrain from cracking his knuckles. No further symptoms or progression of the hand swelling were observed.

## 4 Discussion and conclusion

Pachydermodactyly was first described by Verbov in 1975.^[3]^ It is a rare condition of benign fibromatosis of the digits with fewer than 100 reported cases worldwide. Although its etiology is uncertain, repetitive microtrauma, including frequent finger clicking and hand rubbing, as well as genetic, psychological, and hormonal influences, have been implicated.

Pachydermodactyly is diagnosed based on the clinical symptoms of swelling seen mainly on the radial or ulnar side of the PIP joints, absence of signs of inflammation, normal laboratory tests, and lack of intra-articular or bone involvement revealed by imaging. The hallmark of this disease is absence of pain, discomfort, and morning stiffness. Skin biopsy is usually not required for diagnosis; however, it was performed in our patient, as he had an unusual presentation of unilateral involvement. Very few cases of unilateral involvement have been reported globally.^[4,5]^

The most common histological finding in PDD cases is an increase in dermal collagen along with varying degrees of epidermal hyperkeratosis, acanthosis, mucin deposits, and occasionally, fibroblast proliferation.^[6]^ Our patient had similar findings on skin biopsy, with a hyperkeratotic epidermis and collagenous dermis with an absence of inflammation. The patient was an adolescent boy who presented with swelling in the absence of morning stiffness, localized tenderness, or redness of the involved digits. He frequently cracked his fingers, a habit noticed by his family. Therefore, PDD in our patient may have been linked to the habit of finger cracking.

To help prevent misdiagnosis, several relevant differential diagnoses can be eliminated by careful history taking and examination of the findings. For example, juvenile idiopathic arthritis, Thiemann disease, knuckle pads, pachydermotoperiostosis, progressive pseudorheumatoid dysplasia, and xanthomatous deposits have been reported in the differential diagnosis of PDD in previous studies.^[7]^

Our patient was misdiagnosed with juvenile idiopathic arthritis, a disease characterized by erythematous changes, edema, warmth, and persistent pain in several joints for >6 weeks.^[8]^ However, after reviewing the patient's history, conducting further investigations, and searching the literature, the diagnosis was revised to PDD.

Knuckle pads commonly affect the dorsal and extensor muscles of the fingers and tend to be localized.^[9]^ Thiemann disease commonly manifests in adolescence and is characterized by vascular necrosis of the proximal interphalangeal joints, finger deformities, sclerotic epiphyses, and shorter phalanges on hand radiographs.^[10]^ Progressive pseudorheumatoid dysplasia is a rare hereditary illness.^[11]^ Pachydermotoperiostosis is an autosomal dominant disorder characterized by periostitis and new bone growth.^[12]^

No therapy for PDD has yet been widely acknowledged. Previous reports have indicated that cessation of triggering activities, such as those associated with repetitive microtrauma, can be helpful. Topical or intralesional corticosteroid injections were administered at varying degrees of success.^[13]^ In rare cases, surgical excision has been explored for cosmetic reasons.^[14]^

In conclusion, our case exemplifies the clinical characteristics of PDD despite unilateral involvement. Normal blood test findings and characteristic fusiform swelling in the lateral aspects of the PIP joints without structural involvement and histological findings confirmed the diagnosis. Early recognition of this rare benign condition helps physicians appropriately reassure the patient and his parents without exposing them to unnecessary therapy. The patient was educated to change his habits, stop cracking his fingers, and was informed about the benign nature of his disease.

## Acknowledgments

The author would like to express his gratitude to Dr Hala Yousif, a pathologist at King Salman bin Abdelaziz Medical City, for her generous help with the histopathological interpretation. Additionally, he would like to extend his gratitude to Editage for the English language editing.

## Author contributions

**Conceptualization:** Roaa Aljohani.

**Data curation:** Roaa Aljohani.

**Formal analysis:** Roaa Aljohani.

**Writing – original draft:** Roaa Aljohani.

**Writing – review & editing:** Roaa Aljohani.
